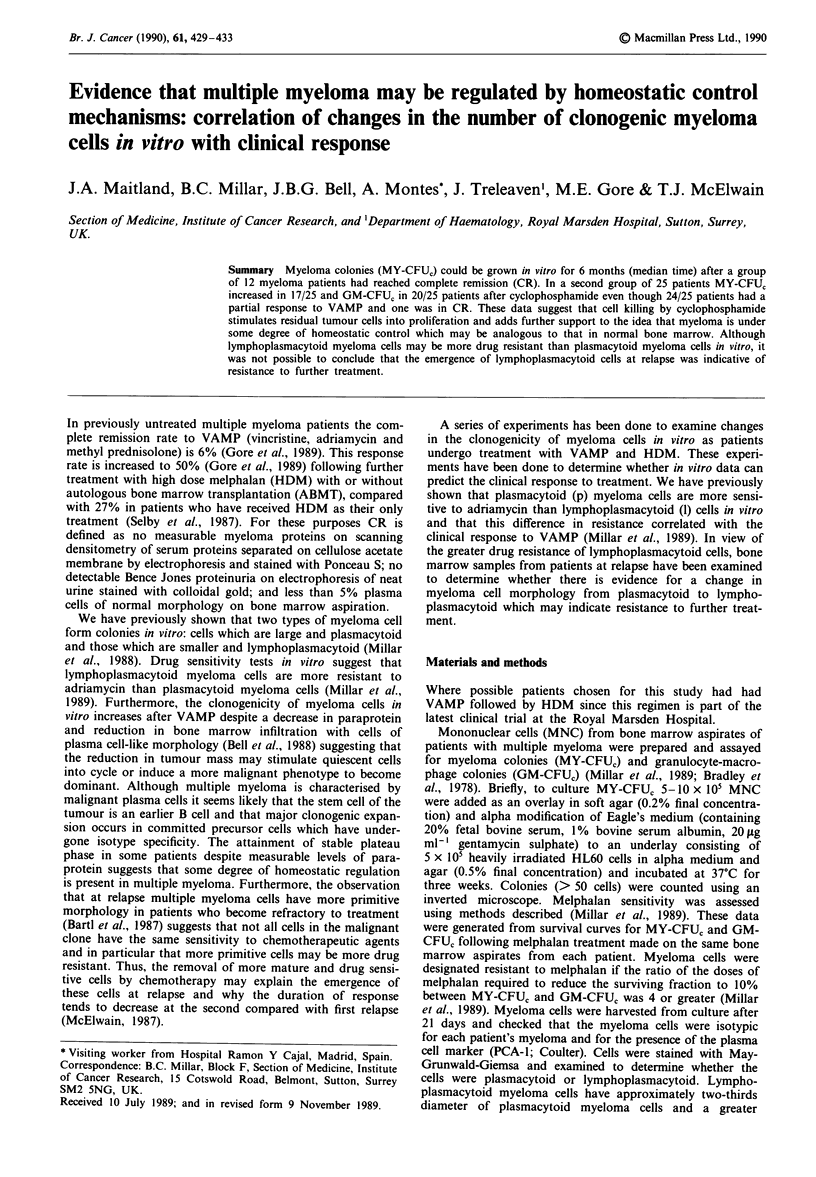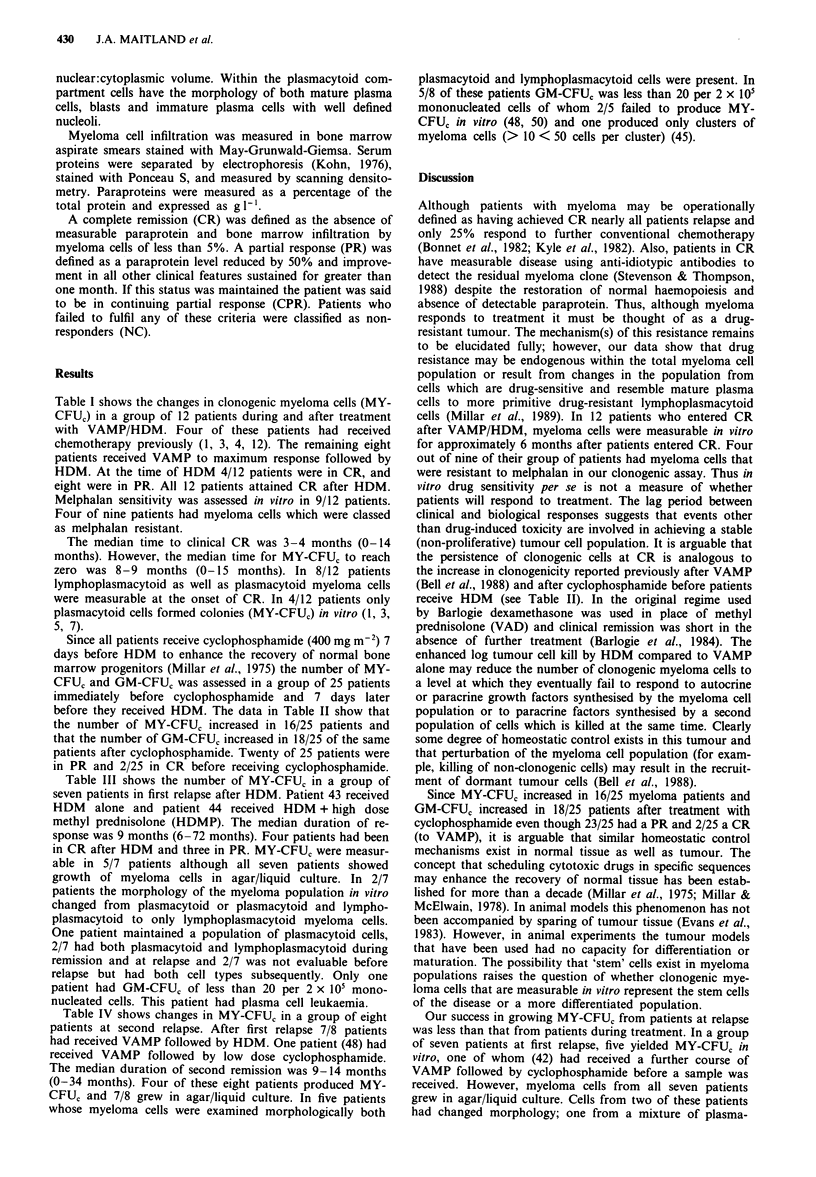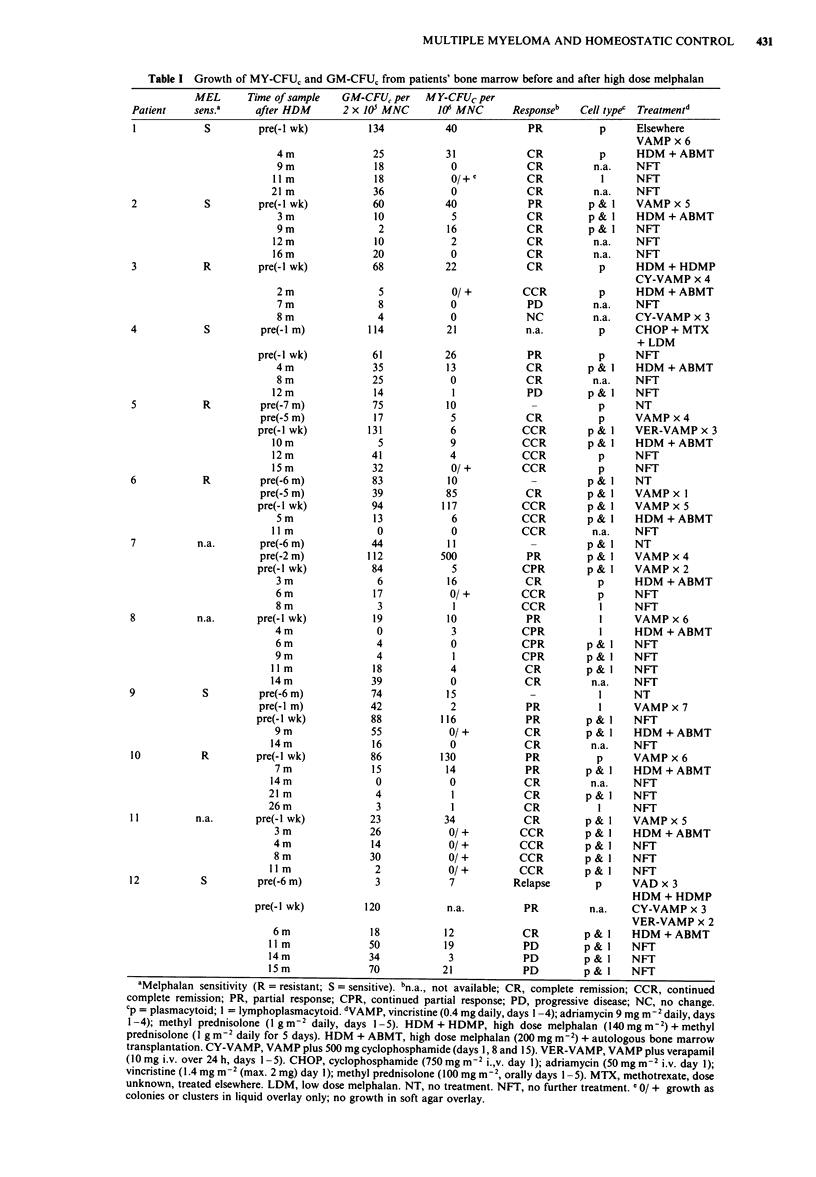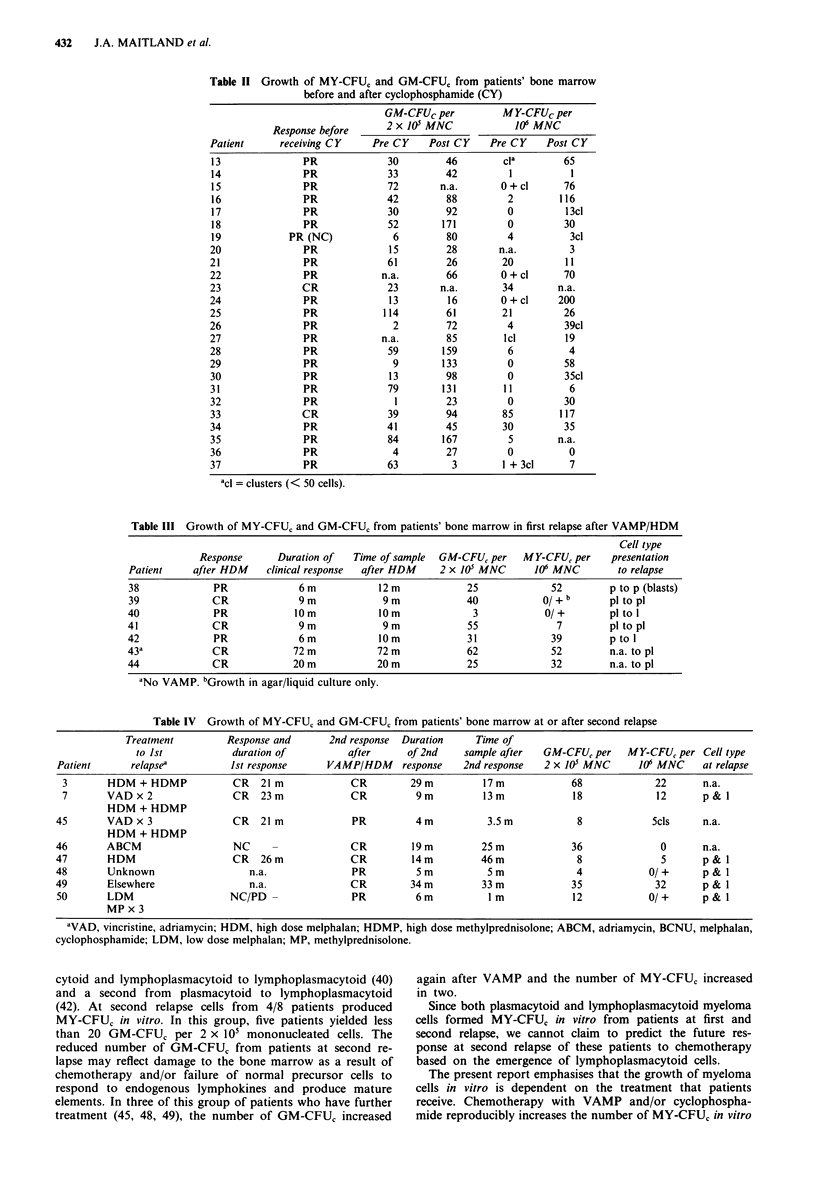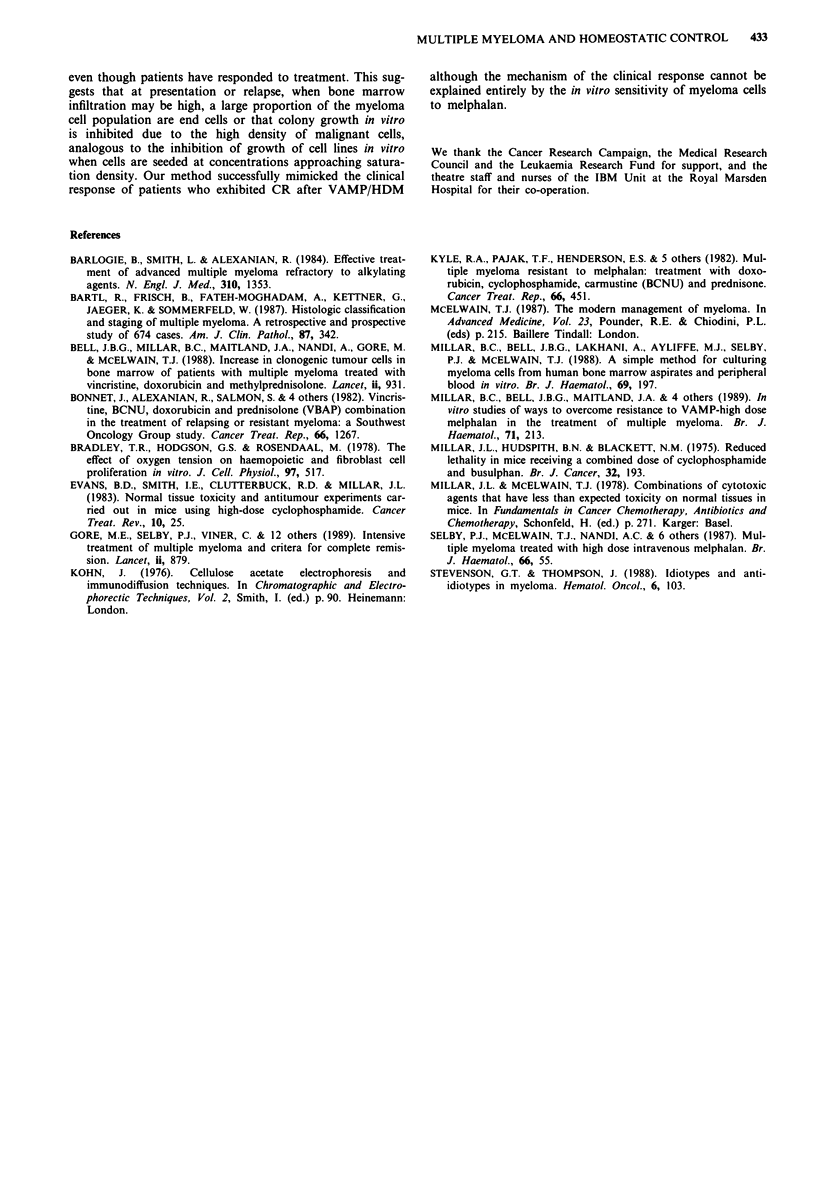# Evidence that multiple myeloma may be regulated by homeostatic control mechanisms: correlation of changes in the number of clonogenic myeloma cells in vitro with clinical response.

**DOI:** 10.1038/bjc.1990.94

**Published:** 1990-03

**Authors:** J. A. Maitland, B. C. Millar, J. B. Bell, A. Montes, J. Treleaven, M. E. Gore, T. J. McElwain

**Affiliations:** Section of Medicine, Royal Marsden Hospital, Sutton, Surrey, UK.

## Abstract

Myeloma colonies (MY-CFUc) could be grown in vitro for 6 months (median time) after a group of 12 myeloma patients had reached complete remission (CR). In a second group of 25 patients MY-CFUc increased in 17/25 and GM-CFUc in 20/25 patients after cyclophosphamide even though 24/25 patients had a partial response to VAMP and one was in CR. These data suggest that cell killing by cyclophosphamide stimulates residual tumour cells into proliferation and adds further support to the idea that myeloma is under some degree of homeostatic control which may be analogous to that in normal bone marrow. Although lymphoplasmacytoid myeloma cells may be more drug resistant than plasmacytoid myeloma cells in vitro, it was not possible to conclude that the emergence of lymphoplasmacytoid cells at relapse was indicative of resistance to further treatment.


Br. J. Cancer (1990), 61, 429-433 © Macmillan Press Ltd., 1990

### Evidence that multiple myeloma may be regulated by homeostatic control mechanisms: correlation of changes in the number of clonogenic myeloma cells *in vitro* with clinical response

J.A. Maitland, B.C. Millar, J.B.G. Bell, A. Montes*, J. Treleaven[1], M.E. Gore & T.J. McElwain

*Section of Medicine, Institute of Cancer Research, and [1]Department of Haematology, Royal Marsden Hospital, Sutton, Surrey, UK.*

**Summary** Myeloma colonies (MY-CFU$_c$) could be grown *in vitro* for 6 months (median time) after a group of 12 myeloma patients had reached complete remission (CR). In a second group of 25 patients MY-CFU$_c$ increased in 17/25 and GM-CFU$_c$ in 20/25 patients after cyclophosphamide even though 24/25 patients had a partial response to VAMP and one was in CR. These data suggest that cell killing by cyclophosphamide stimulates residual tumour cells into proliferation and adds further support to the idea that myeloma is under some degree of homeostatic control which may be analogous to that in normal bone marrow. Although lymphoplasmacytoid myeloma cells may be more drug resistant than plasmacytoid myeloma cells *in vitro*, it was not possible to conclude that the emergence of lymphoplasmacytoid cells at relapse was indicative of resistance to further treatment.

In previously untreated multiple myeloma patients the complete remission rate to VAMP (vincristine, adriamycin and methyl prednisolone) is 6% (Gore *et al.*, 1989). This response rate is increased to 50% (Gore *et al.*, 1989) following further treatment with high dose melphalan (HDM) with or without autologous bone marrow transplantation (ABMT), compared with 27% in patients who have received HDM as their only treatment (Selby *et al.*, 1987). For these purposes CR is defined as no measurable myeloma proteins on scanning densitometry of serum proteins separated on cellulose acetate membrane by electrophoresis and stained with Ponceau S; no detectable Bence Jones proteinuria on electrophoresis of neat urine stained with colloidal gold; and less than 5% plasma cells of normal morphology on bone marrow aspiration.

We have previously shown that two types of myeloma cell form colonies *in vitro*: cells which are large and plasmacytoid and those which are smaller and lymphoplasmacytoid (Millar *et al.*, 1988). Drug sensitivity tests *in vitro* suggest that lymphoplasmacytoid myeloma cells are more resistant to adriamycin than plasmacytoid myeloma cells (Millar *et al.*, 1989). Furthermore, the clonogenicity of myeloma cells *in vitro* increases after VAMP despite a decrease in paraprotein and reduction in bone marrow infiltration with cells of plasma cell-like morphology (Bell *et al.*, 1988) suggesting that the reduction in tumour mass may stimulate quiescent cells into cycle or induce a more malignant phenotype to become dominant. Although multiple myeloma is characterised by malignant plasma cells it seems likely that the stem cell of the tumour is an earlier B cell and that major clonogenic expansion occurs in committed precursor cells which have undergone isotype specificity. The attainment of stable plateau phase in some patients despite measurable levels of paraprotein suggests that some degree of homeostatic regulation is present in multiple myeloma. Furthermore, the observation that at relapse multiple myeloma cells have more primitive morphology in patients who become refractory to treatment (Bartl *et al.*, 1987) suggests that not all cells in the malignant clone have the same sensitivity to chemotherapeutic agents and in particular that more primitive cells may be more drug resistant. Thus, the removal of more mature and drug sensitive cells by chemotherapy may explain the emergence of these cells at relapse and why the duration of response tends to decrease at the second compared with first relapse (McElwain, 1987).

* Visiting worker from Hospital Ramon Y Cajal, Madrid, Spain.
Correspondence: B.C. Millar, Block F, Section of Medicine, Institute of Cancer Research, 15 Cotswold Road, Belmont, Sutton, Surrey SM2 5NG, UK.
Received 10 July 1989; and in revised form 9 November 1989.

A series of experiments has been done to examine changes in the clonogenicity of myeloma cells *in vitro* as patients undergo treatment with VAMP and HDM. These experiments have been done to determine whether *in vitro* data can predict the clinical response to treatment. We have previously shown that plasmacytoid (p) myeloma cells are more sensitive to adriamycin than lymphoplasmacytoid (l) cells *in vitro* and that this difference in resistance correlated with the clinical response to VAMP (Millar *et al.*, 1989). In view of the greater drug resistance of lymphoplasmacytoid cells, bone marrow samples from patients at relapse have been examined to determine whether there is evidence for a change in myeloma cell morphology from plasmacytoid to lymphoplasmacytoid which may indicate resistance to further treatment.

### Materials and methods

Where possible patients chosen for this study had had VAMP followed by HDM since this regimen is part of the latest clinical trial at the Royal Marsden Hospital.

Mononuclear cells (MNC) from bone marrow aspirates of patients with multiple myeloma were prepared and assayed for myeloma colonies (MY-CFU$_c$) and granulocyte-macrophage colonies (GM-CFU$_c$) (Millar *et al.*, 1989; Bradley *et al.*, 1978). Briefly, to culture MY-CFU$_c$ $5-10 \times 10^5$ MNC were added as an overlay in soft agar (0.2% final concentration) and alpha modification of Eagle's medium (containing 20% fetal bovine serum, 1% bovine serum albumin, 20 $\mu$g ml$^{-1}$ gentamycin sulphate) to an underlay consisting of $5 \times 10^5$ heavily irradiated HL60 cells in alpha medium and agar (0.5% final concentration) and incubated at 37°C for three weeks. Colonies (> 50 cells) were counted using an inverted microscope. Melphalan sensitivity was assessed using methods described (Millar *et al.*, 1989). These data were generated from survival curves for MY-CFU$_c$ and GM-CFU$_c$ following melphalan treatment made on the same bone marrow aspirates from each patient. Myeloma cells were designated resistant to melphalan if the ratio of the doses of melphalan required to reduce the surviving fraction to 10% between MY-CFU$_c$ and GM-CFU$_c$ was 4 or greater (Millar *et al.*, 1989). Myeloma cells were harvested from culture after 21 days and checked that the myeloma cells were isotypic for each patient's myeloma and for the presence of the plasma cell marker (PCA-1; Coulter). Cells were stained with May-Grunwald-Giemsa and examined to determine whether the cells were plasmacytoid or lymphoplasmacytoid. Lymphoplasmacytoid myeloma cells have approximately two-thirds diameter of plasmacytoid myeloma cells and a greater nuclear:cytoplasmic volume. Within the plasmacytoid compartment cells have the morphology of both mature plasma cells, blasts and immature plasma cells with well defined nucleoli.

Myeloma cell infiltration was measured in bone marrow aspirate smears stained with May-Grunwald-Giemsa. Serum proteins were separated by electrophoresis (Kohn, 1976), stained with Ponceau S, and measured by scanning densitometry. Paraproteins were measured as a percentage of the total protein and expressed as $g\,l^{-1}$.

A complete remission (CR) was defined as the absence of measurable paraprotein and bone marrow infiltration by myeloma cells of less than 5%. A partial response (PR) was defined as a paraprotein level reduced by 50% and improvement in all other clinical features sustained for greater than one month. If this status was maintained the patient was said to be in continuing partial response (CPR). Patients who failed to fulfil any of these criteria were classified as non-responders (NC).

### Results

Table I shows the changes in clonogenic myeloma cells (MY-$CFU_c$) in a group of 12 patients during and after treatment with VAMP/HDM. Four of these patients had received chemotherapy previously (1, 3, 4, 12). The remaining eight patients received VAMP to maximum response followed by HDM. At the time of HDM 4/12 patients were in CR, and eight were in PR. All 12 patients attained CR after HDM. Melphalan sensitivity was assessed *in vitro* in 9/12 patients. Four of nine patients had myeloma cells which were classed as melphalan resistant.

The median time to clinical CR was 3–4 months (0–14 months). However, the median time for MY-$CFU_c$ to reach zero was 8–9 months (0–15 months). In 8/12 patients lymphoplasmacytoid as well as plasmacytoid myeloma cells were measurable at the onset of CR. In 4/12 patients only plasmacytoid cells formed colonies (MY-$CFU_c$) *in vitro* (1, 3, 5, 7).

Since all patients receive cyclophosphamide (400 mg $m^{-2}$) 7 days before HDM to enhance the recovery of normal bone marrow progenitors (Millar *et al.*, 1975) the number of MY-$CFU_c$ and GM-$CFU_c$ was assessed in a group of 25 patients immediately before cyclophosphamide and 7 days later before they received HDM. The data in Table II show that the number of MY-$CFU_c$ increased in 16/25 patients and that the number of GM-$CFU_c$ increased in 18/25 of the same patients after cyclophosphamide. Twenty of 25 patients were in PR and 2/25 in CR before receiving cyclophosphamide.

Table III shows the number of MY-$CFU_c$ in a group of seven patients in first relapse after HDM. Patient 43 received HDM alone and patient 44 received HDM + high dose methyl prednisolone (HDMP). The median duration of response was 9 months (6–72 months). Four patients had been in CR after HDM and three in PR. MY-$CFU_c$ were measurable in 5/7 patients although all seven patients showed growth of myeloma cells in agar/liquid culture. In 2/7 patients the morphology of the myeloma population *in vitro* changed from plasmacytoid or plasmacytoid and lymphoplasmacytoid to only lymphoplasmacytoid myeloma cells. One patient maintained a population of plasmacytoid cells, 2/7 had both plasmacytoid and lymphoplasmacytoid during remission and at relapse and 2/7 was not evaluable before relapse but had both cell types subsequently. Only one patient had GM-$CFU_c$ of less than 20 per $2 \times 10^5$ mononucleated cells. This patient had plasma cell leukaemia.

Table IV shows changes in MY-$CFU_c$ in a group of eight patients at second relapse. After first relapse 7/8 patients had received VAMP followed by HDM. One patient (48) had received VAMP followed by low dose cyclophosphamide. The median duration of second remission was 9–14 months (0–34 months). Four of these eight patients produced MY-$CFU_c$ and 7/8 grew in agar/liquid culture. In five patients whose myeloma cells were examined morphologically both plasmacytoid and lymphoplasmacytoid cells were present. In 5/8 of these patients GM-$CFU_c$ was less than 20 per $2 \times 10^5$ mononucleated cells of whom 2/5 failed to produce MY-$CFU_c$ *in vitro* (48, 50) and one produced only clusters of myeloma cells (> 10 < 50 cells per cluster) (45).

### Discussion

Although patients with myeloma may be operationally defined as having achieved CR nearly all patients relapse and only 25% respond to further conventional chemotherapy (Bonnet *et al.*, 1982; Kyle *et al.*, 1982). Also, patients in CR have measurable disease using anti-idiotypic antibodies to detect the residual myeloma clone (Stevenson & Thompson, 1988) despite the restoration of normal haemopoiesis and absence of detectable paraprotein. Thus, although myeloma responds to treatment it must be thought of as a drug-resistant tumour. The mechanism(s) of this resistance remains to be elucidated fully; however, our data show that drug resistance may be endogenous within the total myeloma cell population or result from changes in the population from cells which are drug-sensitive and resemble mature plasma cells to more primitive drug-resistant lymphoplasmacytoid cells (Millar *et al.*, 1989). In 12 patients who entered CR after VAMP/HDM, myeloma cells were measurable *in vitro* for approximately 6 months after patients entered CR. Four out of nine of their group of patients had myeloma cells that were resistant to melphalan in our clonogenic assay. Thus *in vitro* drug sensitivity *per se* is not a measure of whether patients will respond to treatment. The lag period between clinical and biological responses suggests that events other than drug-induced toxicity are involved in achieving a stable (non-proliferative) tumour cell population. It is arguable that the persistence of clonogenic cells at CR is analogous to the increase in clonogenicity reported previously after VAMP (Bell *et al.*, 1988) and after cyclophosphamide before patients receive HDM (see Table II). In the original regime used by Barlogie dexamethasone was used in place of methyl prednisolone (VAD) and clinical remission was short in the absence of further treatment (Barlogie *et al.*, 1984). The enhanced log tumour cell kill by HDM compared to VAMP alone may reduce the number of clonogenic myeloma cells to a level at which they eventually fail to respond to autocrine or paracrine growth factors synthesised by the myeloma cell population or to paracrine factors synthesised by a second population of cells which is killed at the same time. Clearly some degree of homeostatic control exists in this tumour and that perturbation of the myeloma cell population (for example, killing of non-clonogenic cells) may result in the recruitment of dormant tumour cells (Bell *et al.*, 1988).

Since MY-$CFU_c$ increased in 16/25 myeloma patients and GM-$CFU_c$ increased in 18/25 patients after treatment with cyclophosphamide even though 23/25 had a PR and 2/25 a CR (to VAMP), it is arguable that similar homeostatic control mechanisms exist in normal tissue as well as tumour. The concept that scheduling cytotoxic drugs in specific sequences may enhance the recovery of normal tissue has been established for more than a decade (Millar *et al.*, 1975; Millar & McElwain, 1978). In animal models this phenomenon has not been accompanied by sparing of tumour tissue (Evans *et al.*, 1983). However, in animal experiments the tumour models that have been used had no capacity for differentiation or maturation. The possibility that 'stem' cells exist in myeloma populations raises the question of whether clonogenic myeloma cells that are measurable *in vitro* represent the stem cells of the disease or a more differentiated population.

Our success in growing MY-$CFU_c$ from patients at relapse was less than that from patients during treatment. In a group of seven patients at first relapse, five yielded MY-$CFU_c$ *in vitro*, one of whom (42) had received a further course of VAMP followed by cyclophosphamide before a sample was received. However, myeloma cells from all seven patients grew in agar/liquid culture. Cells from two of these patients had changed morphology; one from a mixture of plasma-

**Table I** Growth of MY-CFU$_c$ and GM-CFU$_c$ from patients' bone marrow before and after high dose melphalan

| Patient | *MEL sens.*[a] | *Time of sample after HDM* | *GM-CFU$_c$ per $2 \times 10^5$ MNC* | *MY-CFU$_C$ per $10^6$ MNC* | *Response*[b] | *Cell type*[c] | *Treatment*[d] |
|---|---|---|---|---|---|---|---|
| 1 | S | pre(-1 wk) | 134 | 40 | PR | p | Elsewhere VAMP × 6 |
| | | 4 m | 25 | 31 | CR | p | HDM + ABMT |
| | | 9 m | 18 | 0 | CR | n.a. | NFT |
| | | 11 m | 18 | 0/+[e] | CR | l | NFT |
| | | 21 m | 36 | 0 | CR | n.a. | NFT |
| 2 | S | pre(-1 wk) | 60 | 40 | PR | p & l | VAMP × 5 |
| | | 3 m | 10 | 5 | CR | p & l | HDM + ABMT |
| | | 9 m | 2 | 16 | CR | p & l | NFT |
| | | 12 m | 10 | 2 | CR | n.a. | NFT |
| | | 16 m | 20 | 0 | CR | n.a. | NFT |
| 3 | R | pre(-1 wk) | 68 | 22 | CR | p | HDM + HDMP CY-VAMP × 4 |
| | | 2 m | 5 | 0/ + | CCR | p | HDM + ABMT |
| | | 7 m | 8 | 0 | PD | n.a. | NFT |
| | | 8 m | 4 | 0 | NC | n.a. | CY-VAMP × 3 |
| 4 | S | pre(-1 m) | 114 | 21 | n.a. | p | CHOP + MTX + LDM |
| | | pre(-1 wk) | 61 | 26 | PR | p | NFT |
| | | 4 m | 35 | 13 | CR | p & l | HDM + ABMT |
| | | 8 m | 25 | 0 | CR | n.a. | NFT |
| | | 12 m | 14 | 1 | PD | p & l | NFT |
| 5 | R | pre(-7 m) | 75 | 10 | – | p | NT |
| | | pre(-5 m) | 17 | 5 | CR | p | VAMP × 4 |
| | | pre(-1 wk) | 131 | 6 | CCR | p & l | VER-VAMP × 3 |
| | | 10 m | 5 | 9 | CCR | p & l | HDM + ABMT |
| | | 12 m | 41 | 4 | CCR | p | NFT |
| | | 15 m | 32 | 0/ + | CCR | p | NFT |
| 6 | R | pre(-6 m) | 83 | 10 | – | p & l | NT |
| | | pre(-5 m) | 39 | 85 | CR | p & l | VAMP × 1 |
| | | pre(-1 wk) | 94 | 117 | CCR | p & l | VAMP × 5 |
| | | 5 m | 13 | 6 | CCR | p & l | HDM + ABMT |
| | | 11 m | 0 | 0 | CCR | n.a. | NFT |
| 7 | n.a. | pre(-6 m) | 44 | 11 | – | p & l | NT |
| | | pre(-2 m) | 112 | 500 | PR | p & l | VAMP × 4 |
| | | pre(-1 wk) | 84 | 5 | CPR | p & l | VAMP × 2 |
| | | 3 m | 6 | 16 | CR | p | HDM + ABMT |
| | | 6 m | 17 | 0/ + | CCR | p | NFT |
| | | 8 m | 3 | 1 | CCR | l | NFT |
| 8 | n.a. | pre(-1 wk) | 19 | 10 | PR | l | VAMP × 6 |
| | | 4 m | 0 | 3 | CPR | l | HDM + ABMT |
| | | 6 m | 4 | 0 | CPR | p & l | NFT |
| | | 9 m | 4 | 1 | CPR | p & l | NFT |
| | | 11 m | 18 | 4 | CR | p & l | NFT |
| | | 14 m | 39 | 0 | CR | n.a. | NFT |
| 9 | S | pre(-6 m) | 74 | 15 | – | l | NT |
| | | pre(-1 m) | 42 | 2 | PR | l | VAMP × 7 |
| | | pre(-1 wk) | 88 | 116 | PR | p & l | NFT |
| | | 9 m | 55 | 0/ + | CR | p & l | HDM + ABMT |
| | | 14 m | 16 | 0 | CR | n.a. | NFT |
| 10 | R | pre(-1 wk) | 86 | 130 | PR | p | VAMP × 6 |
| | | 7 m | 15 | 14 | PR | p & l | HDM + ABMT |
| | | 14 m | 0 | 0 | CR | n.a. | NFT |
| | | 21 m | 4 | 1 | CR | p & l | NFT |
| | | 26 m | 3 | 1 | CR | l | NFT |
| 11 | n.a. | pre(-1 wk) | 23 | 34 | CR | p & l | VAMP × 5 |
| | | 3 m | 26 | 0/ + | CCR | p & l | HDM + ABMT |
| | | 4 m | 14 | 0/ + | CCR | p & l | NFT |
| | | 8 m | 30 | 0/ + | CCR | p & l | NFT |
| | | 11 m | 2 | 0/ + | CCR | p & l | NFT |
| 12 | S | pre(-6 m) | 3 | 7 | Relapse | p | VAD × 3 HDM + HDMP |
| | | pre(-1 wk) | 120 | n.a. | PR | n.a. | CY-VAMP × 3 VER-VAMP × 2 |
| | | 6 m | 18 | 12 | CR | p & l | HDM + ABMT |
| | | 11 m | 50 | 19 | PD | p & l | NFT |
| | | 14 m | 34 | 3 | PD | p & l | NFT |
| | | 15 m | 70 | 21 | PD | p & l | NFT |

[a]Melphalan sensitivity (R = resistant; S = sensitive). [b]n.a., not available; CR, complete remission; CCR, continued complete remission; PR, partial response; CPR, continued partial response; PD, progressive disease; NC, no change. [c]p = plasmacytoid; l = lymphoplasmacytoid. [d]VAMP, vincristine (0.4 mg daily, days 1–4); adriamycin 9 mg $m^{-2}$ daily, days 1–4); methyl prednisolone (1 g $m^{-2}$ daily, days 1–5). HDM + HDMP, high dose melphalan (140 mg $m^{-2}$) + methyl prednisolone (1 g $m^{-2}$ daily for 5 days). HDM + ABMT, high dose melphalan (200 mg $m^{-2}$) + autologous bone marrow transplantation. CY-VAMP, VAMP plus 500 mg cyclophosphamide (days 1, 8 and 15). VER-VAMP, VAMP plus verapamil (10 mg i.v. over 24 h, days 1–5). CHOP, cyclophosphamide (750 mg $m^{-2}$ i.,v. day 1); adriamycin (50 mg $m^{-2}$ i.v. day 1); vincristine (1.4 mg $m^{-2}$ (max. 2 mg) day 1); methyl prednisolone (100 mg $m^{-2}$, orally days 1–5). MTX, methotrexate, dose unknown, treated elsewhere. LDM, low dose melphalan. NT, no treatment. NFT, no further treatment. [e]0/ + growth as colonies or clusters in liquid overlay only; no growth in soft agar overlay.

**Table II** Growth of $MY\text{-}CFU_c$ and $GM\text{-}CFU_c$ from patients' bone marrow before and after cyclophosphamide (CY)

| Patient | Response before receiving CY | $GM\text{-}CFU_c$ per $2 \times 10^5$ MNC | | $MY\text{-}CFU_c$ per $10^6$ MNC | |
|---|---|---|---|---|---|
| | | Pre CY | Post CY | Pre CY | Post CY |
| 13 | PR | 30 | 46 | cl[a] | 65 |
| 14 | PR | 33 | 42 | 1 | 1 |
| 15 | PR | 72 | n.a. | 0 + cl | 76 |
| 16 | PR | 42 | 88 | 2 | 116 |
| 17 | PR | 30 | 92 | 0 | 13cl |
| 18 | PR | 52 | 171 | 0 | 30 |
| 19 | PR (NC) | 6 | 80 | 4 | 3cl |
| 20 | PR | 15 | 28 | n.a. | 3 |
| 21 | PR | 61 | 26 | 20 | 11 |
| 22 | PR | n.a. | 66 | 0 + cl | 70 |
| 23 | CR | 23 | n.a. | 34 | n.a. |
| 24 | PR | 13 | 16 | 0 + cl | 200 |
| 25 | PR | 114 | 61 | 21 | 26 |
| 26 | PR | 2 | 72 | 4 | 39cl |
| 27 | PR | n.a. | 85 | 1cl | 19 |
| 28 | PR | 59 | 159 | 6 | 4 |
| 29 | PR | 9 | 133 | 0 | 58 |
| 30 | PR | 13 | 98 | 0 | 35cl |
| 31 | PR | 79 | 131 | 11 | 6 |
| 32 | PR | 1 | 23 | 0 | 30 |
| 33 | CR | 39 | 94 | 85 | 117 |
| 34 | PR | 41 | 45 | 30 | 35 |
| 35 | PR | 84 | 167 | 5 | n.a. |
| 36 | PR | 4 | 27 | 0 | 0 |
| 37 | PR | 63 | 3 | 1 + 3cl | 7 |

[a]cl = clusters (< 50 cells).

**Table III** Growth of $MY\text{-}CFU_c$ and $GM\text{-}CFU_c$ from patients' bone marrow in first relapse after VAMP/HDM

| Patient | Response after HDM | Duration of clinical response | Time of sample after HDM | $GM\text{-}CFU_c$ per $2 \times 10^5$ MNC | $MY\text{-}CFU_c$ per $10^6$ MNC | Cell type presentation to relapse |
|---|---|---|---|---|---|---|
| 38 | PR | 6 m | 12 m | 25 | 52 | p to p (blasts) |
| 39 | CR | 9 m | 9 m | 40 | 0/ +[b] | pl to pl |
| 40 | PR | 10 m | 10 m | 3 | 0/ + | pl to l |
| 41 | CR | 9 m | 9 m | 55 | 7 | pl to pl |
| 42 | PR | 6 m | 10 m | 31 | 39 | p to l |
| 43[a] | CR | 72 m | 72 m | 62 | 52 | n.a. to pl |
| 44 | CR | 20 m | 20 m | 25 | 32 | n.a. to pl |

[a]No VAMP. [b]Growth in agar/liquid culture only.

**Table IV** Growth of $MY\text{-}CFU_c$ and $GM\text{-}CFU_c$ from patients' bone marrow at or after second relapse

| Patient | Treatment to 1st relapse[a] | Response and duration of 1st response | 2nd response after VAMP/HDM | Duration of 2nd response | Time of sample after 2nd response | $GM\text{-}CFU_c$ per $2 \times 10^5$ MNC | $MY\text{-}CFU_c$ per $10^6$ MNC | Cell type at relapse |
|---|---|---|---|---|---|---|---|---|
| 3 | HDM + HDMP | CR 21 m | CR | 29 m | 17 m | 68 | 22 | n.a. |
| 7 | VAD × 2<br>HDM + HDMP | CR 23 m | CR | 9 m | 13 m | 18 | 12 | p & l |
| 45 | VAD × 3<br>HDM + HDMP | CR 21 m | PR | 4 m | 3.5 m | 8 | 5cls | n.a. |
| 46 | ABCM | NC – | CR | 19 m | 25 m | 36 | 0 | n.a. |
| 47 | HDM | CR 26 m | CR | 14 m | 46 m | 8 | 5 | p & l |
| 48 | Unknown | n.a. | PR | 5 m | 5 m | 4 | 0/ + | p & l |
| 49 | Elsewhere | n.a. | CR | 34 m | 33 m | 35 | 32 | p & l |
| 50 | LDM<br>MP × 3 | NC/PD – | PR | 6 m | 1 m | 12 | 0/ + | p & l |

[a]VAD, vincristine, adriamycin; HDM, high dose melphalan; HDMP, high dose methylprednisolone; ABCM, adriamycin, BCNU, melphalan, cyclophosphamide; LDM, low dose melphalan; MP, methylprednisolone.

cytoid and lymphoplasmacytoid to lymphoplasmacytoid (40) and a second from plasmacytoid to lymphoplasmacytoid (42). At second relapse cells from 4/8 patients produced $MY\text{-}CFU_c$ *in vitro*. In this group, five patients yielded less than 20 $GM\text{-}CFU_c$ per $2 \times 10^5$ mononucleated cells. The reduced number of $GM\text{-}CFU_c$ from patients at second relapse may reflect damage to the bone marrow as a result of chemotherapy and/or failure of normal precursor cells to respond to endogenous lymphokines and produce mature elements. In three of this group of patients who have further treatment (45, 48, 49), the number of $GM\text{-}CFU_c$ increased again after VAMP and the number of $MY\text{-}CFU_c$ increased in two.

Since both plasmacytoid and lymphoplasmacytoid myeloma cells formed $MY\text{-}CFU_c$ *in vitro* from patients at first and second relapse, we cannot claim to predict the future response at second relapse of these patients to chemotherapy based on the emergence of lymphoplasmacytoid cells.

The present report emphasises that the growth of myeloma cells *in vitro* is dependent on the treatment that patients receive. Chemotherapy with VAMP and/or cyclophosphamide reproducibly increases the number of $MY\text{-}CFU_c$ *in vitro*

even though patients have responded to treatment. This suggests that at presentation or relapse, when bone marrow infiltration may be high, a large proportion of the myeloma cell population are end cells or that colony growth *in vitro* is inhibited due to the high density of malignant cells, analogous to the inhibition of growth of cell lines *in vitro* when cells are seeded at concentrations approaching saturation density. Our method successfully mimicked the clinical response of patients who exhibited CR after VAMP/HDM although the mechanism of the clinical response cannot be explained entirely by the *in vitro* sensitivity of myeloma cells to melphalan.

We thank the Cancer Research Campaign, the Medical Research Council and the Leukaemia Research Fund for support, and the theatre staff and nurses of the IBM Unit at the Royal Marsden Hospital for their co-operation.

### References

BARLOGIE, B., SMITH, L. & ALEXANIAN, R. (1984). Effective treatment of advanced multiple myeloma refractory to alkylating agents. *N. Engl. J. Med.*, **310**, 1353.

BARTL, R., FRISCH, B., FATEH-MOGHADAM, A., KETTNER, G., JAEGER, K. & SOMMERFELD, W. (1987). Histologic classification and staging of multiple myeloma. A retrospective and prospective study of 674 cases. *Am. J. Clin. Pathol.*, **87**, 342.

BELL, J.B.G., MILLAR, B.C., MAITLAND, J.A., NANDI, A., GORE, M. & MCELWAIN, T.J. (1988). Increase in clonogenic tumour cells in bone marrow of patients with multiple myeloma treated with vincristine, doxorubicin and methylprednisolone. *Lancet*, **ii**, 931.

BONNET, J., ALEXANIAN, R., SALMON, S. & 4 others (1982). Vincristine, BCNU, doxorubicin and prednisolone (VBAP) combination in the treatment of relapsing or resistant myeloma: a Southwest Oncology Group study. *Cancer Treat. Rep.*, **66**, 1267.

BRADLEY, T.R., HODGSON, G.S. & ROSENDAAL, M. (1978). The effect of oxygen tension on haemopoietic and fibroblast cell proliferation *in vitro*. *J. Cell. Physiol.*, **97**, 517.

EVANS, B.D., SMITH, I.E., CLUTTERBUCK, R.D. & MILLAR, J.L. (1983). Normal tissue toxicity and antitumour experiments carried out in mice using high-dose cyclophosphamide. *Cancer Treat. Rev.*, **10**, 25.

GORE, M.E., SELBY, P.J., VINER, C. & 12 others (1989). Intensive treatment of multiple myeloma and critera for complete remission. *Lancet*, **ii**, 879.

KOHN, J. (1976). Cellulose acetate electrophoresis and immunodiffusion techniques. In *Chromatographic and Electrophorectic Techniques, Vol. 2*, Smith, I. (ed.) p. 90. Heinemann: London.

KYLE, R.A., PAJAK, T.F., HENDERSON, E.S. & 5 others (1982). Multiple myeloma resistant to melphalan: treatment with doxorubicin, cyclophosphamide, carmustine (BCNU) and prednisone. *Cancer Treat. Rep.*, **66**, 451.

MCELWAIN, T.J. (1987). The modern management of myeloma. In *Advanced Medicine, Vol. 23*, Pounder, R.E. & Chiodini, P.L. (eds) p. 215. Baillere Tindall: London.

MILLAR, B.C., BELL, J.B.G., LAKHANI, A., AYLIFFE, M.J., SELBY, P.J. & MCELWAIN, T.J. (1988). A simple method for culturing myeloma cells from human bone marrow aspirates and peripheral blood *in vitro*. *Br. J. Haematol.*, **69**, 197.

MILLAR, B.C., BELL, J.B.G., MAITLAND, J.A. & 4 others (1989). *In vitro* studies of ways to overcome resistance to VAMP-high dose melphalan in the treatment of multiple myeloma. *Br. J. Haematol.*, **71**, 213.

MILLAR, J.L., HUDSPITH, B.N. & BLACKETT, N.M. (1975). Reduced lethality in mice receiving a combined dose of cyclophosphamide and busulphan. *Br. J. Cancer*, **32**, 193.

MILLAR, J.L. & MCELWAIN, T.J. (1978). Combinations of cytotoxic agents that have less than expected toxicity on normal tissues in mice. In *Fundamentals in Cancer Chemotherapy, Antibiotics and Chemotherapy*, Schonfeld, H. (ed.) p. 271. Karger: Basel.

SELBY, P.J., MCELWAIN, T.J., NANDI, A.C. & 6 others (1987). Multiple myeloma treated with high dose intravenous melphalan. *Br. J. Haematol.*, **66**, 55.

STEVENSON, G.T. & THOMPSON, J. (1988). Idiotypes and anti-idiotypes in myeloma. *Hematol. Oncol.*, **6**, 103.